# Aberrant activation of latent transforming growth factor-β initiates the onset of temporomandibular joint osteoarthritis

**DOI:** 10.1038/s41413-018-0027-6

**Published:** 2018-09-11

**Authors:** Liwei Zheng, Caixia Pi, Jun Zhang, Yi Fan, Chen Cui, Yang Zhou, Jianxun Sun, Quan Yuan, Xin Xu, Ling Ye, Xu Cao, Xuedong Zhou

**Affiliations:** 1 State Key Laboratory of Oral Diseases, National Clinical Research Center for Oral Diseases, West China Hospital of Stomatology, Sichuan University, Chengdu, China; 2 Department of Orthopaedic Surgery, School of Medicine, Johns Hopkins University, Baltimore, Maryland USA

## Abstract

There is currently no effective medical treatment for temporomandibular joint osteoarthritis (TMJ-OA) due to a limited understanding of its pathogenesis. This study was undertaken to investigate the key role of transforming growth factor-β (TGF-β) signalling in the cartilage and subchondral bone of the TMJ using a temporomandibular joint disorder (TMD) rat model, an ageing mouse model and a Camurati–Engelmann disease (CED) mouse model. In the three animal models, the subchondral bone phenotypes in the mandibular condyles were evaluated by µCT, and changes in TMJ condyles were examined by TRAP staining and immunohistochemical analysis of Osterix and p-Smad2/3. Condyle degradation was confirmed by Safranin O staining, the Mankin and OARSI scoring systems and type X collagen (Col X), p-Smad2/3a and Osterix immunohistochemical analyses. We found apparent histological phenotypes of TMJ-OA in the TMD, ageing and CED animal models, with abnormal activation of TGF-β signalling in the condylar cartilage and subchondral bone. Moreover, inhibition of TGF-β receptor I attenuated TMJ-OA progression in the TMD models. Therefore, aberrant activation of TGF-β signalling could be a key player in TMJ-OA development.

## Osteoarthritis: growth factor signalling drives degeneration of jaw joint

Blocking the activity of a critical growth factor could help treat degenerative disease of the jaw joint, according to experiments in three rodent models. Xuedong Zhou from Sichuan University in Chengdu, China, examined the cartilage and adjoining layer of bone found at the ends of the jawbone in a rat model of temporomandibular joint disorder and in two related mouse models. In all three, the researchers observed tissue abnormalities consistent with what’s seen in humans with osteoarthritis of the jaw joint, a condition with no effective therapeutic options. They showed that transforming growth factor-β, a master regulatory protein, displayed aberrant signalling patterns in these tissues and that blocking this protein’s receptor with a drug attenuated the disease process. The findings help explain what drives jaw joint osteoarthritis — and point to a strategy for treating it.

## Introduction

The most common degenerative condition observed in temporomandibular joint disorder (TMD) is osteoarthritis (OA), which causes severe pain and discomfort on one or both sides of the face.^1,2^ One percent of Hong Kong Chinese individuals have frequent TMD-related jaw pain,^3^ and 14.56% of mainland Chinese patients with TMD exhibit radiographic signs of OA.^4^ Temporomandibular joint OA (TMJ-OA) is a common condition that limits the quality of life of patients.^5,6^ Its prevalence increases with occlusal disorder, which is characterised by marked changes in the condylar cartilage and altered composition and material properties of the cartilage matrix.^7^ Despite previous investigations of the pathomechanism of TMJ-OA, the exact pathogenesis of TMJ-OA remains elusive. More importantly, there is no effective treatment approach for TMJ-OA.

Condylar cartilage, as secondary cartilage, differs from other cartilaginous tissue. It can be easily distinguished as a fibrous layer, a proliferative cell layer, a chondrocytic cell layer and a hypertrophic cell layer according to the cellular characteristics.^8,9^ Microscopically, mandibular condylar cartilage is dissimilar to articular cartilage, especially regarding its constituents. In general, articular cartilage is composed of hyaline cartilage, whereas mandibular condylar cartilage consists largely of fibrocartilage, with thick multilayers composed of several collagen fibre zones.^10^ Subchondral bone provides mechanical support for the overlying articular cartilage and forms the osteochondral unit together with articular cartilage.^11,12^ Cartilage chondrocytes respond to alterations in mechanical loading, which cause changes in subchondral bone.^13,14^ Chondrocyte catabolism can lead to articular cartilage damage in OA.^15^ During the progression of TMJ-OA, a high concentration of type X collagen (Col X) is expressed in articular cartilage with chondrocyte hypertrophy.^16,17^ The thickening of the hypertrophic layer was impressively represented in an OA rodent model.^18^ Condylar cartilage degeneration with calcification and osteophyte formation has also been observed in OA.^19^ Numerous studies have shown that subchondral bone stiffness causes significant mechanical load and breakdown of the overlying cartilage.^20–23^ However, subchondral bone in OA is subjected to a decrease rather than an increase in bone stiffness.^24,25^ In addition, uncoupled bone remodelling by osteoclasts and osteoblasts in subchondral bone contributes to cartilage degeneration, which gradually results in TMJ-OA lesions.^13,26^

In recent years, transforming growth factor-β (TGF-β) has drawn increasing attention in the pathogenesis of OA.^27^ Increasing TGF-β1 signalling activity in knee joint OA causes severe cartilage degeneration, and a high level of TGF-β1 in chondrocytes was detected in models of OA.^28^ The protein levels of TGF-β1 and phosphorylated Smad2/3 (p-Smad2/3) were enhanced in the degenerative cartilage in the TMJ of a genetic form of OA.^29^ Therefore, the interruption of TGF-β1 signalling in articular cartilage leads to articular and condylar cartilage degeneration.^30^ Furthermore, a high concentration of TGF-β1 in subchondral bone was found in an anterior cruciate ligament transection (ACLT) OA mouse model.^31^ The overexpression TGF-β1 signalling in subchondral bone leads to abnormal remodelling and is harmful to cartilage integrity.^32,33^ Thus, TGF-β1 may play a critical role in the oetiology of TMJ-OA.^34^ However, whether inhibition of TGF-β1 signalling in condylar cartilage and subchondral bone can rescue TMJ-OA progression remains to be elucidated.

In the present study, we used three different rodent models (TMD rats, Camurati–Engelmann disease (CED) mice and ageing mice) to represent obvious phenotypes of TMJ-OA and investigated the role of TGF-β1 signalling during TMJ-OA progression. Further analysis was performed to elucidate whether inhibition of TGF-β1 activity attenuates TMJ-OA.

## Results

### Cartilage degeneration in the condyle of TMD and ageing models

**Fig. 1:**
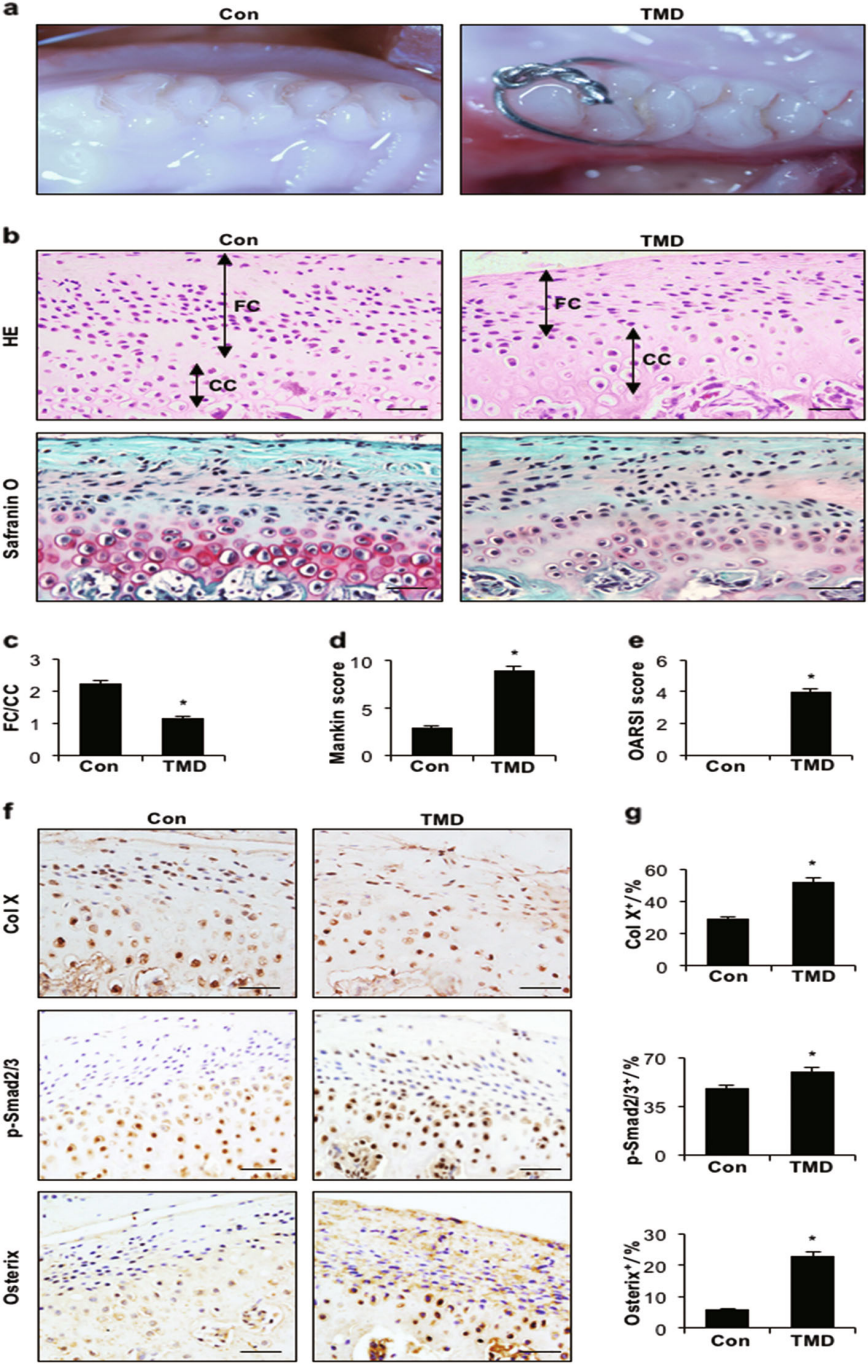
Abnormal occlusion leads to cartilage degeneration in the temporomandibular joint. **a** Representative images of the first molar occlusion relationship in control and TMD rats. **b** HE (top) and Safranin O and fast green (bottom) staining analyses of glycosaminoglycan (red) in sagittal sections of the temporomandibular joint and mandibular condylar cartilage layers (FC fibrocartilage layer, CC: calcified cartilage layer). **c** FC/CC, **d** Mankin and **e** OARSI scores of control and TMD rats. **f** Immunohistochemical analyses of Col X, p-Smad2/3 and Osterix (brown) in the condylar cartilage. **g** Col X-, p-Smad2/3- and Osterix-positive cells (brown) were counted in the cartilage layer. Scale bars = 20 µm. *n* = 6 per group. *P *<* *0.05*, one-way ANOVA followed by Tukey’s test. All data are expressed as the mean ± s.d

The TMD rat model was established by occlusal changes of the first molar (Fig. 1a). Haematoxylin and eosin (HE) staining indicated that the mandibular condylar cartilage was divided into a fibrocartilage layer and a calcified cartilage layer depending on several collagen fibre zones. Compared with rats in the control group, rats in the TMD group showed a significantly decreased fibrocartilage layer and a thinner calcified cartilage layer (Figs. 1b, c). Safranin O staining showed that the distribution of proteoglycans in controls was even and rich, whereas the TMD group exhibited cartilage degradation accompanied by an extensive loss of proteoglycans and a decreased total number of chondrocytes (Fig. 1b). The Mankin and OARSI scores were increased, further confirming the degeneration of articular cartilage in TMD rats compared with controls (Figs. 1d, e). Abnormal, upregulated expression Col X and p-Smad2/3 and an increased number of Osterix-positive cells in the cartilage layer in TMD rats were revealed by immunohistochemistry (Figs. 1f, g).

**Fig. 2:**
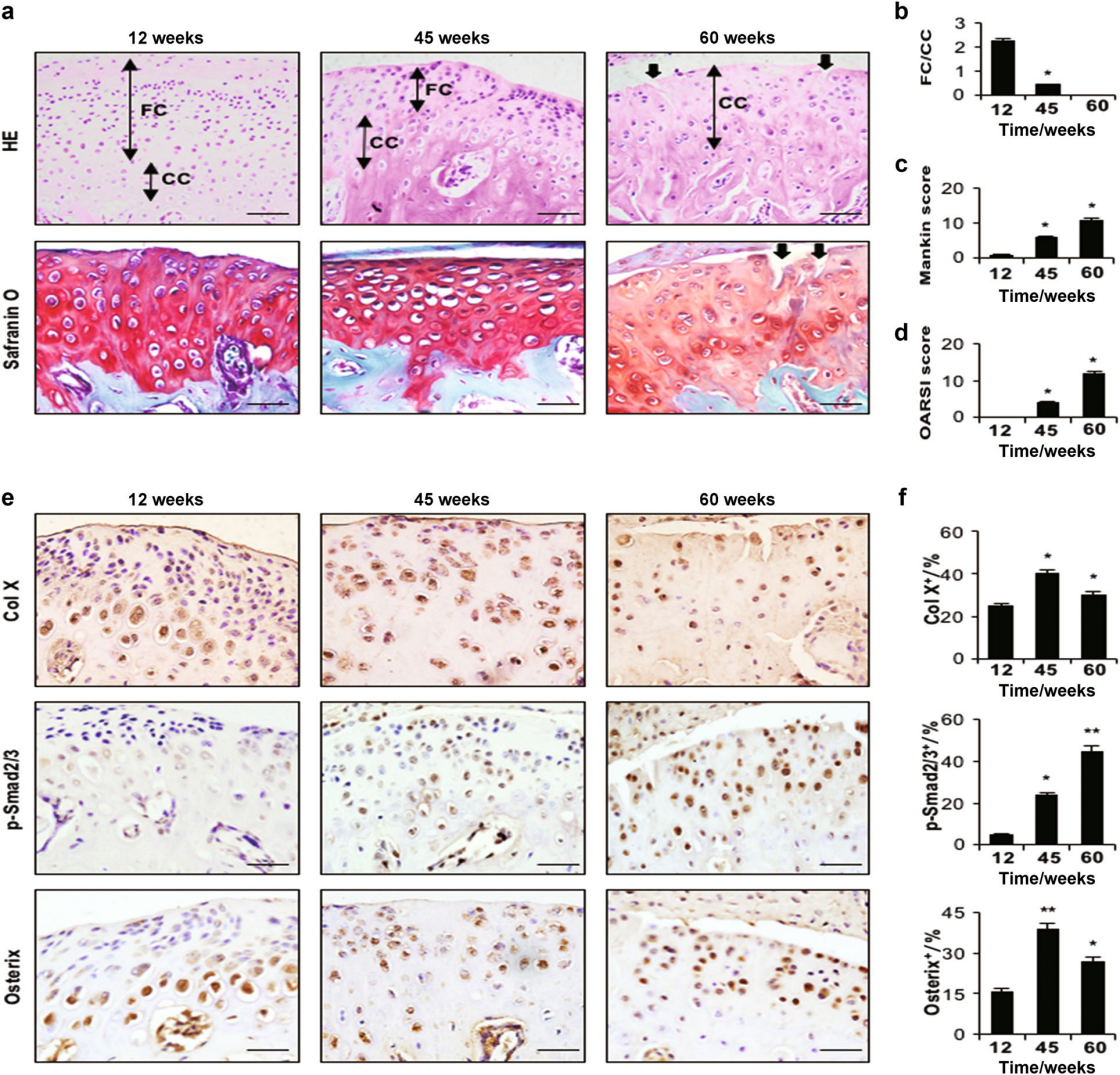
Condylar cartilage degeneration in ageing mice at different time points (12 wks, 45 wks and 60 wks). **a** HE (top) and Safranin O and fast green (bottom) staining analyses of glycosaminoglycan (red), black arrow indicates a crack. Mandibular condylar cartilage cell layers (FC fibrocartilage layer, CC calcified cartilage layer). **b** FC/CC, **c** Mankin and **d** OARSI scores of ageing mice. **e** Immunohistochemical analyses of Col X, p-Smad2/3 and Osterix (brown) in the mandibular condylar cartilage of ageing mice. **f** Col X-, p-Smad2/3- and Osterix-positive cells were counted in the cartilage layer. Scale bars = 20 µm. *n* = 6 per group. *P *<* *0.05*, **P *<* 0.01 one-way ANOVA followed by Tukey’s test. All data are expressed as the mean ± s.d

Age-related changes were also detected in TMJ components. Compared with 12-wk-old mice, the fibrocartilage layer was thinner in 45-wk–old mice, whereas at 60 wks, mice only possessed a calcified cartilage layer with surface fibrillation (cracks) (Figs. 2a, b). Mankin and OARSI scores were higher in 45- and 60-wk-old mice than in 12-wk-old mice (Figs. 2c, d). Moreover, increased Col X and p-Smad2/3 expression and greater numbers of Osterix-positive cells were detected in the cartilage of 45- and 60-wk-old mice (Figs. 2e, f).

### Subchondral bone loss in the TMJ of TMD and ageing models

**Fig. 3:**
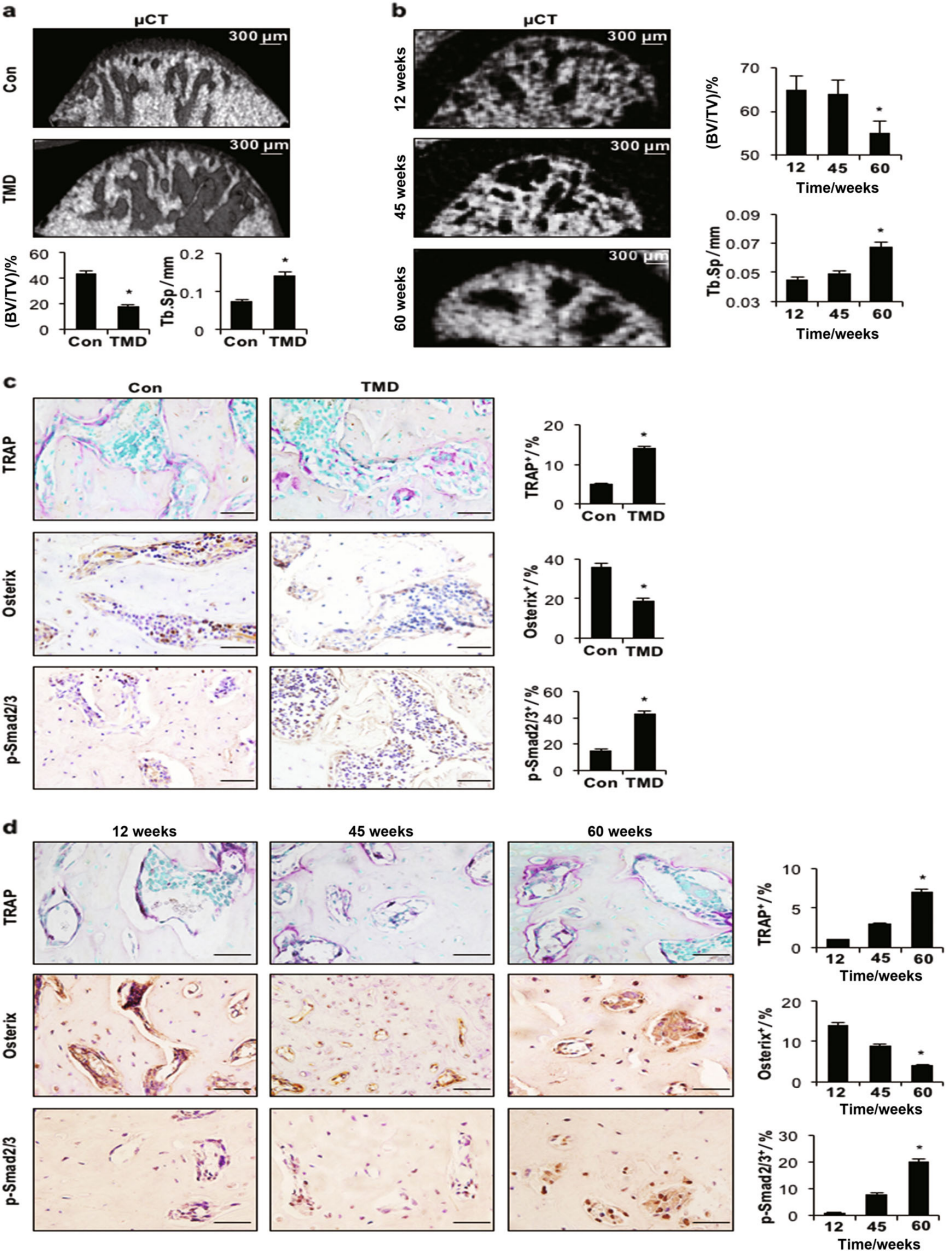
Subchondral bone loss in the temporomandibular joint condyle. **a** Representative µCT images and quantitative analysis showed significant bone loss at 8 wks after surgery in the temporomandibular joint condyle of rats. **b** Representative µCT images and quantitative analysis showed increased bone loss in the temporomandibular joint condyle of ageing mice. Scale bar = 300 μm. BV/TV (%), bone volume fraction; Tb.Sp (mm), trabecular separation. **c**, **d** TRAP staining and immunohistochemical (Osterix and p-Smad2/3) staining of the temporomandibular joint subchondral bone from control and TMD rats (**c**) and ageing mice (**d**). Quantitative analysis of TRAP and IHC staining is shown on the right. Scale bar = 20 μm. *n* = 6 per group. *P *<* 0.05, one-way ANOVA followed by Tukey’s test. All data are expressed as the mean ± s.d

The TMJ subchondral bone of TMD rats was severely altered relative to that in sham-operated controls, as determined by micro-computed tomography (µCT) analysis (Fig. 3a). In the TMD group, bone volume (%, BV/TV) was significantly reduced compared with that in controls, and the trabecular space (Tb.Sp) was significantly increased (Fig. 3a). The number of tartrate-resistant acid phosphatase (TRAP)-positive cells in subchondral bone was significantly increased and the number of Osterix-positive osteoprogenitors was significantly reduced in TMD rats compared with control animals. Similarly, in the ageing mouse model, µCT results revealed that the subchondral bone of 60-wk-old mice was significantly affected, with a significant decrease in bone volume and an increase in Tb.Sp compared with 12- and 45-wk-old animals (Fig. 3b). Moreover, 60-wk-old mice showed a significantly higher percentage of TRAP-positive cells and a lower percentage of Osterix-positive cells in the subchondral bone region than 12- and 45-wk-old mice (Fig. 3d). Notably, a significantly higher number of p-Smad2/3-positive cells was observed in the bone marrow in the TMD group and in ageing mice (60-wk-old mice) than in mice in the respective control groups (Figs. 3c, d).

### Active TGF-β1 in bone induces TMJ-OA

**Fig. 4:**
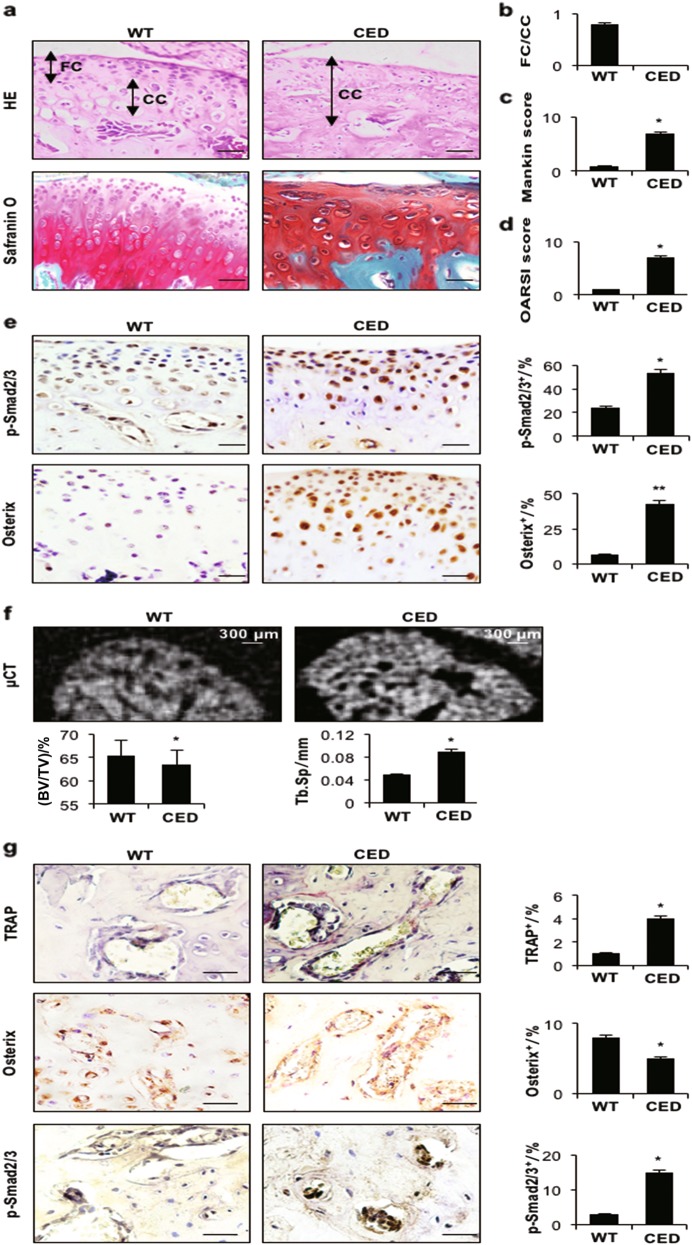
Transgenic activating mutation of TGF-β1 results in a TMJ-OA phenotype. **a** HE (left) and Safranin O and fast green (right) staining analyses of glycosaminoglycan (red) in the condylar cartilage of 10-month-old CED mice and WT littermates. Mandibular condylar cartilage cell layers (FC fibrocartilage layer, CC: calcified cartilage layer). **b** FC/CC, **c** Mankin and **d** OARSI scores of CED mice. **e** Immunohistochemical staining of p-Smad2/3 and Osterix (brown) in the mandibular condylar cartilage of ageing mice (left). p-Smad2/3- and Osterix-positive cells (brown) were counted in the cartilage layer (right). **f** Representative µCT images of the condylar subchondral bone. Quantitative analysis of the structural parameters of subchondral bone is shown on the bottom. **g** TRAP and immunohistochemical (Osterix and p-Smad2/3) staining of the TMJ subchondral bone of CED mice and WT littermates. The percentage of positive cells is shown. Scale bar = 20 μm. *n* = 6 per group. *P *<* 0.05, **P *<* 0.01 one-way ANOVA followed by Tukey’s test. All data are reported as the mean ± s.d

To examine whether high concentrations of active TGF-β1 in subchondral bone initiate TMJ-OA, we used a CED (40-wk-old) activation mutation mouse model in which TGF-β1 is activated after secretion by osteoblastic cells in subchondral bone marrow. HE staining revealed thinner cartilage in CED mice than in control mice. Only calcified cartilage was present in the cartilage of CED mice (Figs. 4a, b). Safranin O staining (Fig. 4a) and higher Mankin and OARSI scores (Figs. 4c, d) revealed significant degeneration of the articular cartilage in CED mice compared with their WT littermates. Moreover, the percentage of p-Smad2/3- and Osterix-positive cells was increased in the cartilage of CED mice (Fig. 4e). µCT images showed an uneven distribution of bone mass in the subchondral bone of the TMJ in CED mice, indicating disrupted bone formation. The volume of subchondral bone (%, BV/TV) was lower, but the Tb.Sp was higher in CED mice than in WT mice (Fig. 4f). In CED mice, the percentage of p-Smad2/3- and TRAP-positive cells in subchondral bone was increased, but the percentage of Osterix-positive cells was lower than in WT mice (Fig. 4g).

### Inhibition of TGF-β signalling attenuates cartilage damage and enhances bone mass in subchondral bone

**Fig. 5:**
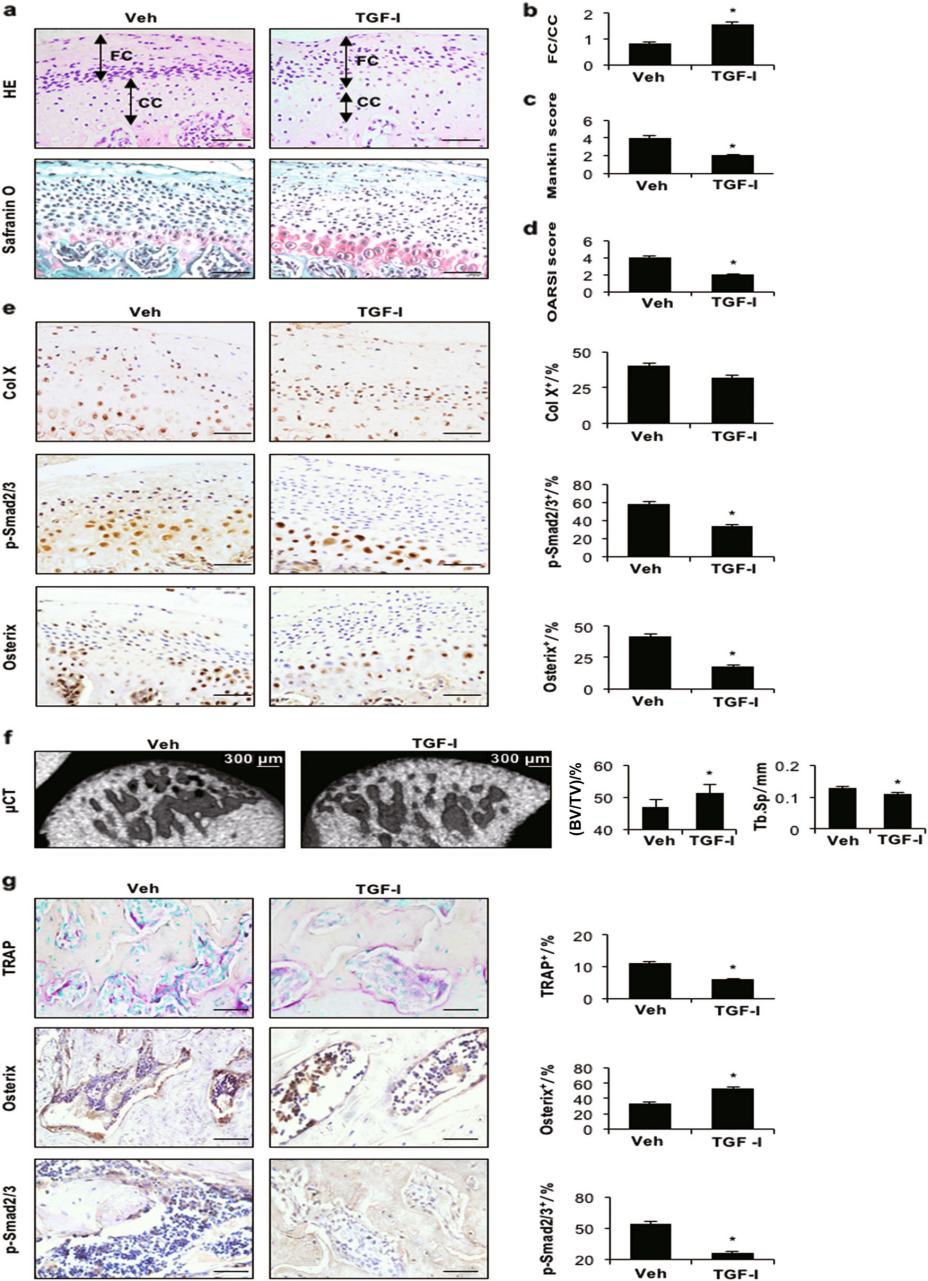
TβRI inhibitor stabilises the subchondral bone microarchitecture in TMJ-OA rats. **a** HE (top) and Safranin O and fast green (bottom) staining analyses of glycosaminoglycan (red) in the TMJ mandibular condyle from TMD rats treated with 1 mg/kg body weight of the TβRI inhibitor daily for 30 days. Mandibular condylar cartilage cell layers (FC fibrocartilage layer, CC calcified cartilage layer). **b** FC/CC, **c** Mankin and **d** OARSI scores of rats. **c** Representative µCT images of the condylar subchondral bone and quantitative analysis of the structural parameters of subchondral bone. Scale bar = 300 μm. **e** Immunohistochemical analyses of Col X, p-Smad2/3 and Osterix (brown) in the mandibular condylar cartilage of TMD rats (left). p-Smad2/3- and Osterix-positive cells (brown) were counted in the cartilage layer (right). **f** Representative µCT images of the condylar subchondral bone. Quantitative analysis of the structural parameters of subchondral bone is shown on the right. **g** TRAP and immunohistochemical (Osterix and p-Smad2/3) staining of the TMJ subchondral bone. Quantitative analysis is shown on the right. Scale bar = 20 μm. *n* = 6 per group. *P *<* 0.05, one-way ANOVA followed by Tukey’s test. All data are reported as the mean ± s.d

To examine the effect of inhibition of TGF-β activity during TMJ-OA progression, we intraperitoneally injected a TGF-β receptor 1 (TβRI) inhibitor or vehicle into TMD rats for 30 days. Upon injection of the TβRI inhibitor (TGF-I), the number of p-Smad2/3-positive cells was dramatically decreased in the cartilage and subchondral bone marrow (Figs. 5b, d), indicating effective inhibition of TGF-β1 signalling. HE staining revealed a thicker fibrocartilage layer in rats injected with the TGF-I than in the vehicle-injected group (Figs. 5a, b). Of note, cartilage degeneration was attenuated in TMD rats injected with the TGF-I, as determined by deeper Safranin O staining and higher Mankin and OARSI scores (Figs. 5c, d). Although there was no difference in the number of Col X-positive cells between the TGF-I group and vehicle treatment group, the percentages of p-Smad2/3- and Osterix-positive cells in the cartilage layer were significantly decreased in the TGF-I group (Fig. 5e). Moreover, injection of the TGF-I caused a significant increase in bone volume and a decrease in Tb.Sp relative to the effects of vehicle treatment (Fig. 5f). In addition, the percentage of TRAP-positive cells was significantly reduced, while that of Osterix-positive cells was increased in subchondral bone marrow after TGF-I injection (Fig. 5g). Thus, inhibition of TGF-β signalling prevented cartilage degeneration and bone resorption in early-stage TMJ-OA.

## Discussion

Severe malocclusion, ageing, psychological stress and skeletal jaw asymmetry cause TMJ-OA.^35^ The limited understanding of its pathogenesis has led to ineffective therapies for restoring the structures of the TMJ with progressive OA. A critical approach to investigating the pathogenesis of TMJ-OA is the use of animal models. However, surgical, mechanical, chemical and gene TMJ-OA models are still unable to mimic its complex clinical conditions.^36–38^ In our study, we used TMD and ageing models to mimic the clinical pathology and progression of TMJ-OA.

Occlusion disturbance is one of the most important factors in TMJ-OA.^39^ Rat models provide adequate amounts of tissue and are easy to handle. Therefore, we established TMD rat models by inducing abnormal mechanical loading on the TMJ. In the initial phase of dental occlusion, there was predominant loss of subchondral bone in the TMJ condyles.^16^ Cartilage degeneration in TMJ-OA is often accompanied by a high OARSI score and abnormally enhanced Col X and matrix metalloproteinase 13 expression.^14,33,34,40^ In our study, Safranin O and HE staining revealed increased thickness of the calcified cartilage layer and cartilage matrix degeneration in the TMD models. Furthermore, bone volume decreased in the subchondral bone of TMD rats. Cartilage damage and the low subchondral bone volume confirmed that we successfully established an early-onset TMJ-OA rat model. Col X is a specific marker of the hypertrophic layer.^41^ Osterix is essential for later-stage endochondral ossification and is involved in OA development.^42^ The expression of Osterix and Col X, which were highly expressed in the hypertrophic layer of cartilage in the TMD groups, was detected in the cartilage. Therefore, high concentrations of Osterix and Col X contribute to the cartilage calcification degeneration in TMD. However, the Osterix level in subchondral bone differed from that in the cartilage of TMD rat models. The number of Osterix-positive osteoprogenitors and osteoblasts in subchondral bone was significantly decreased in TMD rats. These results indicate decreased bone formation ability and uncoupled bone formation and bone resorption in the subchondral bone of TMD rats. p-Smad2/3 was increased in subchondral bone and cartilage disrupted joint microarchitecture, supporting the pathogenesis of OA in TMD.

As a degenerative disease, ageing is a major risk factor for human OA. Ageing-related changes in the musculoskeletal system may contribute to OA development and progression.^43–45^ We established a spontaneous TMJ-OA model using 45- and 60-wk-old C57BL/6J mice. Articular chondrocytes exhibited an age-related decline in proliferation and synthetic capacity.^46^ As age increased, the calcified cartilage layer became thicker. There was a higher number of Osterix- and Col X-positive chondrocytes in 45- and 60-wk-old mice than in 12-wk-old mice. Ageing not only reduced the cartilage thickness but also induced the condylar cartilage to form bone matrix like-tissue.^47^ This result suggests that ageing may be an important factor in cartilage degeneration. In addition, the condylar subchondral bone of ageing mice displayed decreased bone volume, with more TRAP-positive cells and fewer Osterix-positive osteoprogenitor cells in subchondral bone marrow. With ageing, inevitable bone loss occurs, which is frequently the cause of osteoporosis, and inevitable bone and joint degeneration are observed, which often result in osteoarthrosis.^48^ TGF-β levels become excessively high in the blood stream of older people and ageing mice.^49,50^ In the ageing rodent models, p-Smad2/3-positive cells were significantly enhanced in the cartilage and subchondral bone. Therefore, ageing with abnormal TGF-β1 signalling activity aggravated condylar cartilage degeneration and subchondral bone resorption.

OA osteoblasts produced a high level of TGF-β1 signalling, which plays a negative role in cartilage homeostasis and chondrocyte differentiation.^27,51^ The ratio of the TGF-β1 receptor ALK1 to ALK5 was increased in the chondrocytes from aged and OA cartilage.^52^ TGF-β1 plays a pivotal role in bone remodelling, and the TGF-β1 concentration is enhanced in subchondral bone in the ACLT mouse model of OA and in human OA patients.^53^ Interestingly, in both the TMD and ageing TMJ-OA models that exhibited the classic TMJ-OA phenotype, we observed condylar cartilage and subchondral bone with high levels of TGF-β1 signalling activity. These results are consistent with those from human OA studies.^54,55^ High levels of active TGF-β1 signalling occur in the bone marrow microenvironment, leading to abnormal bone remodelling.^31^ TGF-β1 overexpression in subchondral bone caused TMJ mandibular condyle degradation.^33^ In this study, CED mice, an osteoblast-specific mutant TGF-β1 transgenic mouse model, were used to display abnormal active TGF-β1 participating in TMJ-OA progression. Similar to the TMD and ageing animal models, Osterix-positive osteoprogenitor cells and increasing numbers of TRAP-positive cells resulted in uncoupled bone formation in the condylar subchondral bone of CED mice. Cartilage degradation with increased Osterix, p-Smad2/3 and Col X expression were observed in 40-wk-old CED mice relative to age-matched WT mice. These results indicate that TGF-β1 overexpression in subchondral bone induces TMJ-OA. The expression and activity of TGF-β1 signalling significantly increase in the cartilage at the early degenerative stage of OA.^30^ This observation indicates that abnormal activation of TGF-β1 signalling induces cartilage degeneration in TMJ-OA.

The TGF-I was used to inhibit TGF-β1 signalling to rescue cartilage damage induced by OA.^56^ Moreover, the TGF-I ameliorated the abnormal role of high levels of active TGF-β1 in bone formation and directly attenuated Smad2/3 phosphorylation.^57,58^ In our study, injection of the TGF-I significantly decreased the number of p-Smad2/3-positive cells in the condylar cartilage and subchondral bone in the TMD model. The significant increase in Osterix-positive cells in the subchondral bone of the TGF-I group may trigger the bone formation process and lead to increased bone mass in subchondral bone. These results suggest that the TGF-I not only induced an increase in bone volume but also rescued cartilage degeneration.

TMJ-OA is a common degenerative joint disease that limits the quality of life of patients. TMJ-OA oetiology is multifactorial and mainly includes malocclusion, ageing, joint injury and inflammation. We used ageing mice with spontaneous TMJ-OA to examine cartilage and subchondral bone lesions. The TMD models imitated the most common clinical oetiology of TMJ-OA. Furthermore, our results showed that an aberrant level of TGF-β1 signalling in the condylar cartilage induced cartilage degeneration in disordered occlusion animal models, age-related TMJ-OA mice and TGF-β-overexpressing mice. Moreover, significantly high TGF-β1 signalling in subchondral bone contributed to uncoupled bone remodelling in TMJ-OA. Thus, TGF-β1 can be considered a pathogenic factor in the onset of TMJ-OA. Our results suggest that the TGF-β1 concentration may be used as a diagnostic index of human TMJ-OA in clinical applications. Furthermore, it is possible to reverse aberrant TGF-β1 signalling to rescue cartilage degeneration and enhance subchondral bone volume in TMD models. Therefore, targeting the TGF-β1 signalling pathway might be an effective means of treating human TMJ-OA. Biological reagents that inhibit TGF-β1 signalling may be used as drugs to treat TMJ-OA in the clinic.

## Materials and methods

### Ethics statement

The current study was approved by the Ethical Committees of the West China School of Stomatology, Sichuan University, and the State Key Laboratory of Oral Diseases. All experimental methods and procedures were carried out in accordance with the approved guidelines.

### Animal models

Six-week-old male Sprague-Dawley (SD) rats (weighing 160–180 g) were purchased from Chengdu Dossy Biological Technology Co., Ltd. and randomly divided into sham-operated control (Con, operation was performed in a manner similar to the experimental group except without occlusal disorder) and TMD groups (*n* = 18 rats/group). In the experimental group (TMD), disordered occlusion was created by abnormal dental occlusion force based on a previous report.^16,59^ Briefly, an orthodontic ligation silk (0.25-mm diameter) was inserted between the first and second molars, and a ligation silk knot was created on the first molar of the maxillary to induce abnormal mechanical loading on the rat TMJ. TMJ samples from TMD rats were collected 8 wks later. For pharmacological treatment, 4 wks after surgery, animals received intraperitoneal injections of a TGF-I (1 mg/kg, SB-505124, Selleck Chemicals) or an equivalent volume of vehicle (DMSO, Dimethyl sulfoxide) daily for 30 days.^31^ Rats were euthanised 60 days after surgery. Eight-week-old male C57BL/6J mice were obtained from Chengdu Dossy Biological Technology Co., Ltd. and randomly divided into three groups (*n* = 9 mice/group). The mice were maintained in a temperature-controlled room (22 °C) under artificial illumination (lights on from 800 to 1 800 hours) and provided access to food and water ad libitum. During the period of feeding, three mice from each experimental group died. Mice were euthanized at 12 weeks (*n* = 6), 45 weeks (*n* = 6) and 60 weeks (*n* = 6), and TMJ samples of ageing mice were collected.

CED mice (40 weeks old) were obtained from the Animal Facility of the Johns Hopkins University School of Medicine. The CED-derived TGF-β1 mutation, which contains the full-length TGF-β1 with a point mutation (H222D), is specifically expressed by osteoblastic cells driven by a 2.3-kb type I collagen promoter.^60^

### µCT analysis

Samples were imaged on a µCT scanner (μCT50; SCANO, Switzerland). The samples were scanned at a voltage of 50 kVp, a current of 200 µA and a resolution of 5.0 µm per pixel. Sagittal images of the TMJ condyle subchondral bone were used to perform three-dimensional histomorphometric analysis. Two cubic regions of interest (each 0.5 × 0.5 × 0.5 mm^3^) were selected from the middle of the centre and posterior third of the condylar subchondral bone.^16^ Within the selected regions, bone volume fraction (%, BV/TV) and trabecular separation (Tb.Sp) were determined and compared between the experimental and control groups.

### Histological staining

At the time of euthanasia, the TMJ samples of all animals were dissected and fixed in 4% paraformaldehyde overnight. After decalcification in 10% EDTA (pH 7.2–7.4), samples were processed, embedded in paraffin and cut into 5-μm sections using a microtome (Leica, RM2235, Germany). Standard HE staining was used to examine tissue histology. Safranin O and fast green staining was performed to determine proteoglycan changes, and the histological data were further analysed by assessing the Mankin and Osteoarthritis Research Society International (OARSI) scores. The scoring methods were described previously.^61,62^ Semiquantitative Makin and OARSI scores were significantly correlated and positively associated with exercise duration. However, in the rodent model of early OA, the OARSI system was less sensitive than the Mankin sore in reflecting the progression of cartilage change.^63^ We used two scoring systems to semiquantify the severity of TMJ-OA in the ageing and TMD models. TRAP staining was performed using a standard protocol (Sigma-Aldrich).

### Immunohistochemistry

Immunohistochemical analyses of sections of each construct were performed using an Anti-Rabbit/Mouse HRP-DAB Cell & Tissue Staining Kit (R&D Systems, USA). Sections were subjected to epitope recovery in citrate buffer at 99 °C for 30 min. Once room temperature was reached, slides were washed in triethanolamine-buffered saline, and nonspecific immunoglobulin binding was blocked with 5% (V/V) bovine serum albumin for 30 min at room temperature. Sections were incubated overnight at 4 °C with the following primary antibodies: rabbit anti-Osterix (Santa Cruz Biotechnology, 1:100, USA); rabbit anti-Col X (Abcam, 1:100, ab58623) and a TGF-β pathway-specific antibody against p-Smad2/3 (sc-11769, Santa Cruz Biotechnology, 1:100, USA). All sections were incubated with a biotinylated secondary antibody, stained using an R&D HRP-DAB Staining Kit and counterstained with haematoxylin. After mounting, the slides were photographed with an Olympus BX53 microscope (Olympus, Japan). The numbers of Col X-positive cells in the cartilage layer, TRAP-positive osteoclasts, Osterix-positive cells and p-Smad2/3-positive cells in the posterior and middle condylar subchondral bone were determined. The percentages of positive cells in all chondrocytes are shown. All sections were placed onto one slide and processed together under the same conditions.

### Statistical analysis

All experiments were performed independently in triplicate. Comparisons between groups were evaluated with unpaired two-tailed Student’s *t*-test between two groups or with one-way analysis of variance (ANOVA) followed by Tukey’s test for multiple comparisons using SPSS 16.0 software (IBM, Armonk, NY, USA). *P *<* 0.05 was considered to indicate a significant difference between groups.
